# Comprehensive role of prostate‐specific antigen identified with proteomic analysis in prostate cancer

**DOI:** 10.1111/jcmm.15634

**Published:** 2020-07-27

**Authors:** Haoyong Li, Zhe Ma, Zhifei Che, Qi Li, Jinfeng Fan, Zhiyan Zhou, Yaoxi Wu, Yingxia Jin, Peiyu Liang, Xianping Che

**Affiliations:** ^1^ Department of Urology Renmin Hospital of Wuhan University Wuhan China; ^2^ Department of Urology the First Affiliated Hospital of Hainan Medical University Haikou China; ^3^ Central Laboratory Renmin Hospital of Wuhan University Wuhan China; ^4^ Department of Urology The Second Affiliated Hospital of Hainan Medical University Haikou China

**Keywords:** iTRAQ, MCM4, prostate cancer, proteomic analysis, prostate‐specific antigen

## Abstract

Current treatments including androgen deprivation fail to prevent prostate cancer (PrCa) from progressing to castration‐resistant PrCa (CRPC). Accumulating evidence highlights the relevance of prostate‐specific antigen (PSA) in the development and progression of PrCa. The underlying mechanism whereby PSA functions in PrCa, however, has yet been elucidated. We demonstrated that PSA knockdown attenuated tumorigenesis and metastasis of PrCa C4‐2 cells in vitro and in vivo, whereas promoted the apoptosis in vitro. To illuminate the comprehensive role of PSA in PrCa, we performed an isobaric tag for relative and absolute quantitation (iTRAQ)‐based proteomic analysis to explore the proteomic change induced by PSA knockdown. Among 121 differentially expressed proteins, 67 proteins were up‐regulated, while 54 proteins down‐regulated. Bioinformatics analysis was used to explore the mechanism through which PSA exerts influence on PrCa. Protein‐protein interaction analysis showed that PSA may mediate POTEF, EPHA3, RAD51C, HPGD and MCM4 to promote the initiation and progression of PrCa. We confirmed that PSA knockdown induced the up‐regulation of MCM4 and RAD51C, while it down‐regulated POTEF and EPHA3; meanwhile, MCM4 was higher in PrCa para‐cancerous tissue than in cancerous tissue, suggesting that PSA may facilitate the tumorigenesis by mediating MCM4. Our findings suggest that PSA plays a comprehensive role in the development and progression of PrCa.

## 1 INTRODUCTION

Prostate cancer (PrCa) ranks as the most common malignant tumour in men and has become a major cause of cancer‐related death.^1^ Prostate‐specific antigen (PSA), as a member of the kallikrein‐related peptidase (KLK) family of serine proteases, plays a pivotal role in early detection of new cases of PrCa,^2^ and it has been used to monitor the PrCa recurrence following local therapies.^3^ Previous studies, including the European Randomised Study of Screening for PrCa clinical trial, suggest that there exists an association between PSA screening and a decreased risk of PrCa mortality,^4^ which implies a crucial role of PSA in promoting the development and progression of PrCa.^3^

Accumulating evidence highlights the role of PSA in the initiation and progression of PrCa. Free PSA has been found to be linked with expression variations of tumour‐promoter genes and tumour‐suppressor genes in PrCa cell lines.^5^ Veveris‐Lowe and his coworkers showed that PSA and hk4 co‐functioned in the progression of PrCa via inducing the epithelial‐mesenchymal transition (EMT).^6^ In addition, the enzymatic activity of PSA correlates with the tumour growth of PrCa.^3^ Saxena et al^7^ found that PSA trans‐locates to nuclei of androgen‐stimulated PrCa cells with androgen receptor (AR) and further regulated the expression of AR, which affected AR signalling that functions in PrCa development. Nevertheless, the molecular mechanisms of PSA in the development and progression of PrCa have not been fully elucidated.

In the present study, we investigated the role of PSA in PrCa using RNA interference in PrCa C4‐2 cells and explored the proteome‐wide changes induced by PSA knockdown using isobaric tag for relative and absolute quantitation (iTRAQ)‐based LC‐MS/MS analysis. Our findings provide an opportunity to advance our knowledge of the mechanistic role of PSA in PrCa, and provide novel evidence for targeting PSA‐relevant signalling pathway to treat PrCa.

## 2 MATERIALS AND METHODS

### 2.1 Cell line and cell culture

The human PrCa cell line C4‐2 was purchased from ASY Biotechnology Limited Corporation (Wuhan, China). C4‐2 cells were maintained in 90% RPMI 1640 Medium (Cat. No. C11875500BT, Life Technologies, Gibco, USA) supplemented with 10% foetal bovine serum (FBS) (Cat. No. C10010500, Life Technologies, Gibco, USA) and 1% penicillin‐streptomycin (Gibco, USA) at 37°C in a humidified incubator with 5% CO_2_.

### 2.2 Generation of PSA‐specific shRNA‐expressing C4‐2 cells and selection of stably infected monoclonal colonies derived from C4‐2

A shRNA oligonucleotides sequence targeting a region of the PSA cDNA was designed by MISSION^™^ TRC‐Hs 1.0 library (Sigma‐Aldridge, St. Louis, MO).^3^ A shRNA sequence non‐specific to the region of PSA cDNA was designed by Genechem Corporation (Shanghai, China) as a control. The shRNA sequence was as follows: 5′‐GCCTGGAGACATATCACTCAA‐3′; the non‐specific negative control sequence: 5′‐TTCTCCGAACGTGTCACGT‐3′. The shRNA lentivirus expression vector comprising the specific or non‐specific sequence was designed and subsequently packaged in lentiviral particles which were isolated from HEK293T cells (Genechem Corporation, Shanghai, China). Then, the lentiviral particles were utilized to infect C4‐2 cells according to the vendor's recommendations. Briefly, 1000 of C4‐2 cells were planted in a well of 96‐well plate in 200 μL of complete media. After 24 hours, the media was replaced with fresh media. Lentiviral particles and polybrene in certain volumes were added to the cells at a final multiplicity of infection (MOI) of 100. After 12 hours of infection, the media containing particles were replaced with fresh media. After culturing for 72 hours, the cells were digested with trypsin and washed twice with PBS for subsequent suspension in fresh media. The cell concentration was counted, and then, 100 cells were transferred to 10 mL RPMI 1640 media supplemented with 20% FBS in a 100 mm dish (Corning) for further maintaining in the humidified incubator. After about 20 days, when monoclonal colonies derived from C4‐2 cells emerged and were visible by naked eyes, monoclonal colonies expressing GRP among them were transferred to wells of 24‐well plate, respectively, with media supplemented with 20% FBS. When the confluence reached to 90%, those monoclonal colonies were transferred to wells on 6‐well plates filled with fresh compete media for subsequent characterization.

### 2.3 Western blot assay

Cells were digested with trypsin and rinsed twice with PBS and then suspended in radio immunoprecipitation assay buffer (RIPA buffer) (Beyotime Biotechnology, Shanghai, China) mixed with protease inhibitors phenylmethanesulphonyl fluoride (PMSF) (Beyotime Biotechnology) and another protease inhibitor cocktail (Servicebio Corporation, Wuhan, China) for at least 30 minutes on ice. After lysing, cell lysates were further crushed using Ultrasonic Cell Crusher prior to centrifugation in a microcentrifuge at 12 000***g*** for 10 minutes at 4°C and collection of supernatant. A protein quantification assay was performed using bicinchoninic acid (BCA) protein assay kit (Beyotime Biotechnology) to determine the protein concentration. Then, the supernatant was mixed with 1/4 volume of 5 × SDS‐PAGE Sample Loading Buffer (Beyotime Biotechnology). The protein‐loading buffer mixtures were boiled at 95°C for five minutes to denature proteins. Equivalent amount (30 μg) of proteins for cell samples was loaded into wells of a 12% sodium dodecyl sulphate‐polyacrylamide gel electrophoresis (SDS‐PAGE) gel (Biotechwell, Shanghai, China), along with molecular weight marker (Cat. No. 26617, Page Ruler Prestained Protein Ladder, Thermo). Electrophoresis was performed for separation of proteins prior to a transferring process in which proteins were transferred from the gel to polyvinylidene difluoride (PVDF) membranes (Cat. No. IPFL00010, Millipore, USA). The PVDF membranes were blocked for one hour with 5% non‐fat milk (Cat. No. 232100, BD Biosciences, USA) at room temperature and subsequently incubated with primary antibodies for PSA or β‐Actin at 4°C overnight. Secondary antibodies were then incubated for one and a half hours at room temperature. After rinsed thoroughly within TBST, protein bands were scanned and visualized using Odyssey infrared imaging system (LI‐COR Biosciences, USA). Experiments were repeated three times, and two monoclonal colonies in which PSA expression was most largely reduced were picked up depending on data analysis using ImageJ software. Primary antibodies were purchased from Cell Signaling Technology (USA): PSA/KLK3 rabbit monoclonal antibody (Cat. No. 5877), MCM4 rabbit monoclonal antibody (Cat. No. 12973, CST, USA), POTEF antibody (Cat. No. PA5‐24128, Thermo Fisher, USA), RAD51C (Cat. No. sc‐390697, SANTA CRUZ, USA), EPHA3 (Cat. No. 8793, CST, USA) and β‐Actin mouse monoclonal antibody (Cat. No. 3700, CST, USA). Secondary antibodies were anti‐rabbit IgG (Cat. No. 5151, CST, USA) and antimouse IgG (Cat. No. 926‐32210, LI‐COR Biosciences, USA).

### 2.4 Cell proliferation assay

Cell proliferation assay was performed utilizing the Cell Counting Kit‐8 (CCK‐8) following the manufacturer's instructions (Cat. No. CK04, Dojindo, Japan). In brief, C4‐2‐derived shNC cells and two shPSA monoclonal colonies were inoculated in a 96‐well plate to investigate the effect of endogenous PSA on cell proliferation, while 2000 C4‐2 cells were planted in a 96‐well plate to investigate the effect of exogenous PSA. At time points of 0 hour, 24 hour, 48 hour and 72 hour, 10 μL WST reagents were added per well, and the cells were incubated for 2 hours. Then, the absorbance was scaled at 450 nm using a multilabel counter (PerkinElmer, Singapore).

### 2.5 Cell migration and invasion assays

Migration assay was performed using 24‐well Transwell plates with 8 μm pore size (Cat. No. 3422, Corning, USA) and bottoms of chambers lacking a covering of Matrigel Matrix (Cat. No. 356234, BD Biosciences, USA), whereas invasion assay was conducted using the Transwell plates coated with Matrigel Matrix at a 1:8 dilution ratio. The cells at a concentration of 1 × 10^5^ cells in 100 μL non‐FBS RPMI 1640 were seeded in the upper chamber and 600 μL RPMI 1640 containing 20% FBS was seeded in the lower chamber. After incubating for 48 hours at 37°C with 5% CO_2_, cells that had migrated or invaded through the bottom of chambers were fixed with 4% paraformaldehyde for 15 minutes prior to staining with 0.05% Crystal Violet for 30 minutes. The number of migration or invasion cells was counted under a microscope.

### 2.6 Apoptosis assay

Apoptosis assay was performed using Phycoerythrin (PE) Annexin V Apoptosis Detection Kit I (Cat. No. 559763, BD Pharmingen^™^, USA) following the recommended procedure provided by the manufacturer. Briefly, cells were seeded in 6‐well plates first and once attachment, the medium was replaced with FBS‐free medium. After starvation for 24 hours, the cells were digested with EDTA‐free trypsin and neutralized by complete medium. All cells in supernate and in attachment were collected via centrifugation in a microcentrifuge at 2500 *g* for 5 minutes. Cell pellets were rinsed twice with cold PBS and resuspended in 1X Binding Buffer. Then, 100 μL of cells at a concentration of 1 × 10^6^ cells/mL were transferred to a 5‐mL culture tube. Five microlitres of PE Annexin V and five microlitres of 7‐aminoactinomycin D (7‐AAD) were added into the tube, and then, the cells were gently vortexed and incubated for 15 minutes at 25°C in the dark. Finally, the tube was mixed with 400 μL of 1X Binding Buffer and the cells were analysed by a flow cytometry (BD FACSCalibur^™^, USA).

### 2.7 In vivo tumorigenesis and metastasis assays

To evaluate the effect of decreased PSA expression on tumorigenesis and metastasis in murine models, in vivo tumorigenesis and metastasis assays were performed as described previously.^3^, ^8^ In tumorigenesis assay, cells were collected via treatment with 0.25% trypsin‐EDTA solution and rinsed in Hank's balanced salt solution (HBSS). After suspended in HBSS containing 60% Matrigel Matrix, cells at a concentration of 5 × 10^6^ cells per 100 μL solution were then injected subcutaneously into male nude athymic BALB/c mice (six‐week‐old) (n = 7). Tumour measurements were performed with callipers at 2‐day intervals. Tumour volume (in mm^3^) was calculated by the formula 0.5 × (long diameter) × (short diameter)^2^.^8^ In metastasis assay, cells at a concentration of 2 × 10^6^ cells per 200 μL HBSS were injected intravenously into the tail vein of nude mice (n = 7). All the mice were killed by CO_2_ overdose transiently after 19 days. Afterwards, lung samples were subjected to haematoxylin‐eosin staining. Metastatic tumour area in the field of microscope were calculated using Image‐Pro Plus 6.0. All animal experiments were carried out in accordance with the National Institutes of Health guide for the care and use of Laboratory animals, as well as approved by the Medical Ethical Committee of the Renmin Hospital of Wuhan University.

### 2.8 Sample preparation for iTRAQ labelling

A total of 2 × 10^7^ C4‐2‐derived shNC cells or one monoclonal colony (in which PSA expression was most extremely inhibited, n = 3) was collected in a tube. The samples were resuspended and thoroughly solubilized by lysis buffer and then sonicated in ice. The samples were bathed in boiling water for 15 minutes, followed by centrifugation at 14 000***g*** for 15 minutes. Debris was discarded, and protein concentration was measured using BCA assay.

A total of 30 μL of protein solution of each sample was mixed with dithiothreitol (DTT) at a final concentration of 100 mΜ, bathed in boiling water for 5 minutes and then chilled at room temperature. After addition of 200 μL UA buffer (8Μ urea, 150 mΜ Tris‐HCl, pH 8.5), the proteins were enriched using 30‐kDa centrifugal filter (Sartorius, Germany) at 14 000***g*** for 15 minutes, which was repeated twice. Addition of 100 μL iodoacetamide (IAA) buffer (100 mΜ IAA in UA) was performed for protein alkylation prior to a vortex for one minute. After being incubated in dark for 30 minutes, the samples were centrifuged at 14 000***g*** for 15 minutes. Addition of 100 μL UA buffer was followed by centrifugation at 14 000***g*** for 15 minutes, and this process was repeated twice. After addition of 100 μL diluted dissolution buffer (AB SCIEX, USA) for ten times, the samples were centrifuged at 14 000***g*** for 15 minutes and repeated twice. The samples were added 40 μL trypsin buffer (4 μg trypsin in 40 μL dissolution buffer) and then vortexed for 1 minute. After standing for 16 hours, the samples were centrifuged at 14 000***g*** for 15 minutes for filtrate. After addition of 40 μL of dissolution buffer with ten times of dilution, the samples were centrifuged at 14 000***g*** for 15 minutes. Quantitation of peptides was performed using NanoDrop 2000 (Thermo scientific, USA).

### 2.9 iTRAQ Labelling and high pH RP fractionation

The peptides of 100 μg taken from each sample were labelled using iTRAQ reagents (AB SCIEX, USA) following the manufacturer's protocol. The labelled peptides were mixed, and then, chromatography was conducted with Agilent 1260 Infinity II Liquid Chromatography System (Agilent, USA). Labelled peptide mixtures were reconstituted within high pH RP buffer A (10 mΜ HCOONH_4_, 5% acetonitrile, pH 10.0) and loaded by a manual injector onto a 4.6 mm × 100 mm XBridge Peptide BEH C18 column (Thermo Scientific, USA). The peptides were eluted at a flow rate of 1 mL/min. The gradient was 0% high pH RP buffer B (10 mΜ HCOONH4, 85% acetonitrile, pH 10.0) for 5 minutes, 0%‐7% buffer B for 25 minutes, 7%‐40% buffer B for 30 minutes, 40%‐100% buffer B for 65 minutes and 100% buffer B for 70 minutes. The absorbance value at 214 nm was detected, and fractions were collected at intervals of one minute, amounting to 36 fractions. Those fractions were pooled for each sample and lyophilized by a vacuum centrifuge. Fractions for each sample were then resuspended in 0.1% formic acid (FA) (Buffer A of the mobile phrase for LC‐MS/MS).

### 2.10 LC‐MS/MS analysis, protein identification and quantification

The sample was separated using EASY‐nLC 1200 Liquid Chromatography System(Thermo Scientific, USA) and loaded onto 50 µm × 15 cm Acclaim PepMap RSLC column (Thermo Scientific, USA) at a flow rate of 300 nL/min The gradient condition was 0%‐6% buffer B (0.1% FA, 80% acetonitrile) for five minutes, 6%‐28% buffer B for 45 minutes, 28%‐38% buffer B for 50 minutes, 38%‐100% buffer B for 50 minutes and hold in 100% buffer B for 60 minutes. After separation, the sample was detected using Q Exactive Plus mass spectrometer (Thermo Scientific, USA). The mass over charge (m/z) range of the precursor ion was set from 350 to 1850 in the MS scan, while MS spectra were obtained at 70 000 resolution. Raw MS data were subjected to identification using Mascot 2.5. The parameters were used in protein identification when using Mascot 2.5 as follow: 2 as Max Missed Cleavages, ±20 ppm as Precursor Mass Tolerance, 0.1 Da as Fragment Mass Tolerance, iTRAQ‐8‐plex as Modification Groups from Quan Method, Oxidation (M), Acetyl (Protein N‐term), Deamidated (NQ) as Dynamic modifications, Carbamidomethyl (C) as Static modifications, decoy as Database pattern and ≤0.01 as Peptide FDR. In quantification analysis, the protein ratios were calculated as the median of only unique peptides of the protein using Proteome Discoverer 2.1. All those peptide ratios were normalized by the median protein ratio for ensuring the median protein ratio should be 1 after the normalization.

### 2.11 Bioinformatics analysis

To visualize the overall proteome‐wide expression differences between shNC cells and PSA‐silenced cells, hierarchical cluster analysis was performed using Multiple Experiment Viewer (MeV) software, which could generate a heatmap.

Gene Ontology (GO) functional annotation and Kyoto Encyclopedia of Genes and Genomes (KEGG) enrichment analysis of 121 differentially expressed proteins were performed using Cytoscape with the ClueGO^9^ and CluePedia^10^ plugins based on GO and KEGG databases. To discover the biological protein‐protein associations, 121 proteins were subjected to formation of interaction networks using Search Tool for the Retrieval of Interacting Genes (STRING, https://string‐db.org/) database,^11^ and afterwards, the generated STRING protein‐protein interaction (PPI) network was imported into Cytoscape with the cytoHubba ^12^ plugin containing one topological analysis method named Maximal Clique Centrality (MCC) to predict essential proteins from the existing STRING PPI network.

### 2.12 Patient selection and tissue preparation

Totally, 27 men were enrolled who had been diagnosed with PrCa of acinar type between January 2016 and December 2017 in Renmin Hospital of Wuhan. Gleason score ≥ 7, being free of chemotherapy and radiotherapy were considered in filtration. Cancer tissue specimens and adjacent tissue specimens, identified by experienced pathologist, were embedded in paraffin, sectioned and then subjected to immunohistochemical staining. This work was carried out in accordance with The Code of Ethics of the World Medical Association and approved by the Ethics Committee of Renmin Hospital of Wuhan University. Informed consent was obtained for experimentation with human subjects.

### 2.13 Immunohistochemistry

Expression level of MCM4 in tissue specimens was measured by IHC following the IHC protocol developed by R&D Systems (https://www.rndsystems.com) Briefly, tissue sections were deparaffinized with xylene and alcohol and then rehydrated by buffer water. Endogenous peroxidase activity was quenched using peroxidase blocking reagent and retrieval solution was used for retrieving antigen. Primary antibody applied was MCM4 Rabbit monoclonal antibody (1:100 dilution, Cat. No. 12973, CST, US). Staining of tissue was visualized under a microscope.

### 2.14 Statistical analysis

All experiments were performed in three biological replicates at least. Data were showed as mean ± standard deviation (SD). One‐way ANOVA or Student's *t* test was applied for the PSA‐induced phenotype experiments in vitro and in vivo using SPSS 23.0 (IBM, USA). Wilcoxon rank test was used in the statistical analysis of clinicopathological samples. Statistical analyses of bioinformatics were based on built‐in methods in databases. The level of statistical significance was defined as *P* < .05.

## 3 RESULTS

### 3.1 Verification of PSA knockdown efficiency in C4‐2‐derived monoclonal colonies

**Figure 1:**
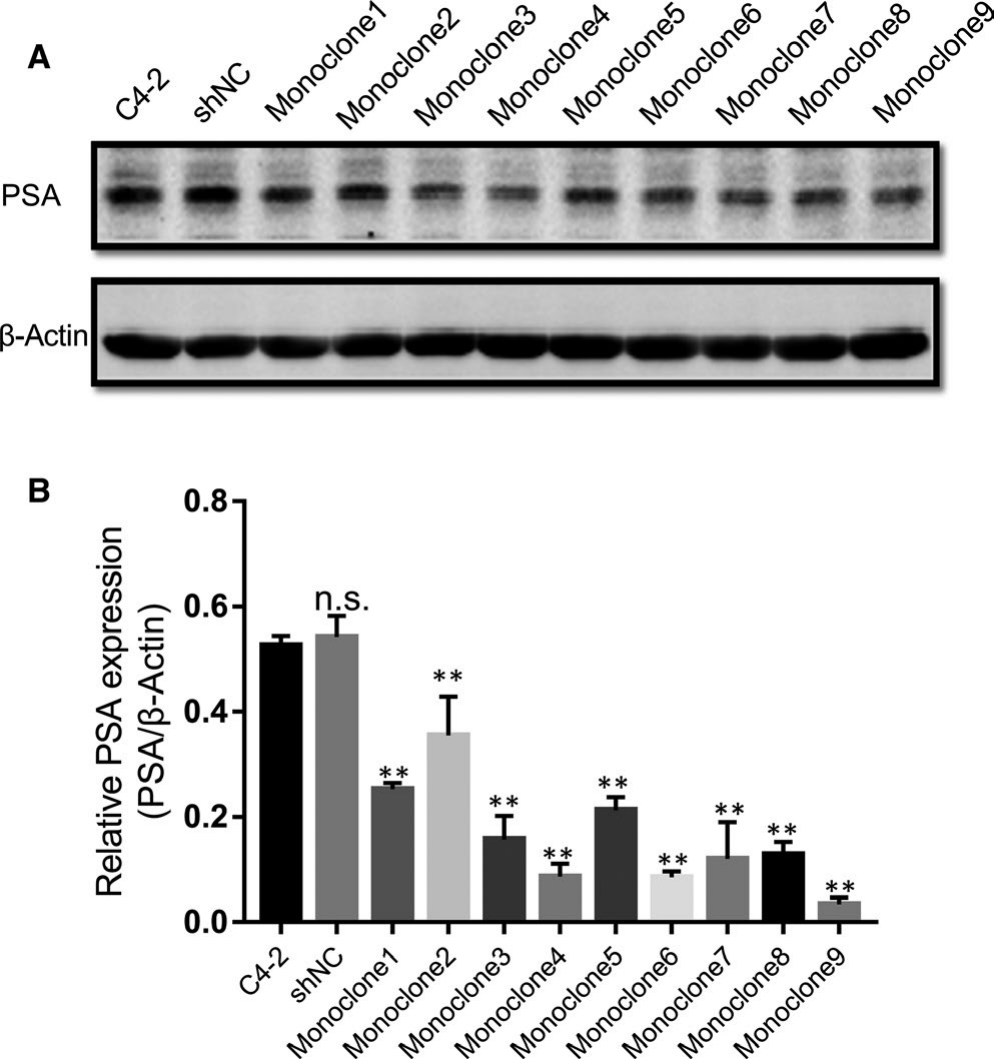
Specific down‐regulation of PSA expression by shRNA. Nine monoclonal colonies derived from C4‐2 cells infected with lentiviral particles containing the PSA‐specific shRNA were lysed for cell lysates, and PSA levels of them were subsequently measured via Western Blot, as well as C4‐2‐derived shNC cells, which were infected with a control construct. C4‐2 cell lysates as blank control. Means and 95% confidence intervals displayed for experiments repeated three times. *P < .05, **P < .01, n.s. not significant. Error bars represent SD of the mean of three independent experiments. shNC, C4‐2‐derived cells infected with scrambled control; Monoclone1‐9, monoclonal colonies of C4‐2‐derived cells infected with PSA‐targeting shRNA construct

By infecting C4‐2 cells with a packaged lentivirus expressing PSA‐targeting shRNA, we acquired nine monoclonal colonies. The Western blot semi‐quantitation assay demonstrated that two monoclonal colonies, Monoclone6 and Monoclone9, possessed the lowest expression levels of PSA, and in specific, Monoclone9 produced less PSA protein than Monoclone6 (Figure 1). Specifically, compared with shRNA cells, 83% and 93% PSA production were reduced in Monoclone6 (*P* < .001) and Monoclone9 (*P* < .001), respectively. Monoclone6 cells, Monoclone9 cells and shNC cells were used for verifying the biological effect of PSA on CRPC C4‐2 cells.

### 3.2 PSA knockdown inhibits proliferation of C4‐2 cells in vitro

**Figure 2:**
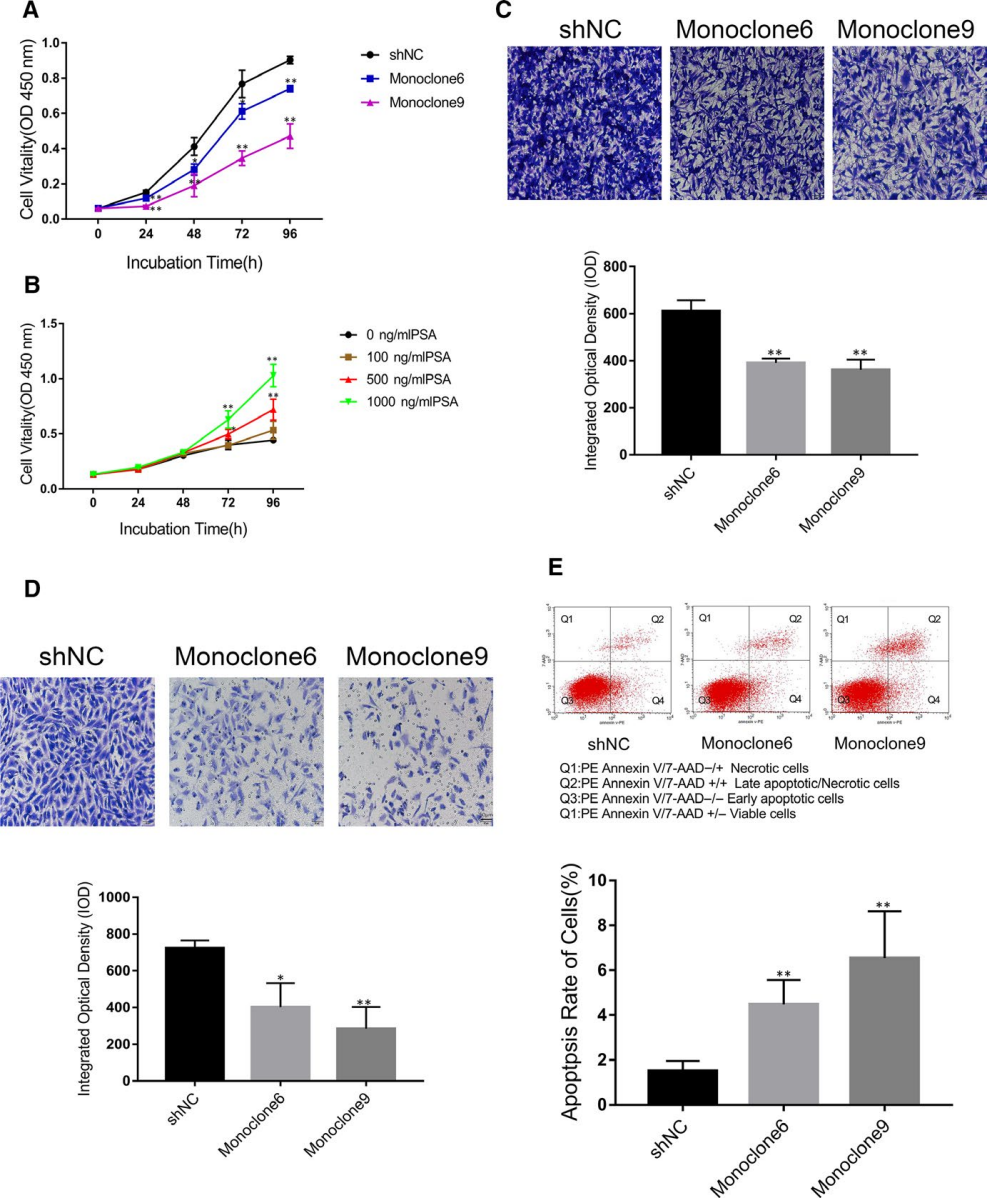
Effects of varying of PSA expression on prostate cancer cells proliferation, migration, invasion and apoptosis in vitro. A, C4‐2‐derived populations expressing PSA‐specific shRNA (the colourful lines), or a control construct (the black line), were subjected to the CCK‐8 assay. Means and 95% confidence intervals are displayed. B, Effects of exogenous PSA in varying concentrations on C4‐2 cells. C, Effects of silencing of PSA expression on the migration ability of C4‐2‐derived cells measured using cell migration assay. D, Effects of inhibiting of PSA expression on the invasion ability of C4‐2‐derived cells were measured using cell invasion assay. E, Changes in apoptosis rate of the cells accompanied by the lowering of PSA expression were measured via PE Annexin V/7‐AAD assay. * indicates *P* < .05, and ** for *P* < .01. Scale bar = 50 μm. Error bars denote SD of the mean of three independent experiments

Williams et al^3^ demonstrated that decreased endogenous PSA hindered PrCa LNCaP cell growth in vitro and in vivo, whereas Bindukumar et al^5^ showed that purified free PSA treatment reduced PC‐3M tumour growth. To confirm the effects of PSA in PrCa, we performed CCK‐8 assay to measure the proliferation of C4‐2‐derived Monoclone6, Monoclone9 and shNC cells. As shown in Figure 2A, knockdown of PSA decelerated the proliferation of Monoclone6 and Monoclone9 cells, and Monoclone9 exhibited the more obvious deceleration in growth rate at 24 hours, 48 hours, 72 hours and 96 hours, suggesting that PSA contributing to the progression of PrCa.

### 3.3 *Exogenous PSA promotes the proliferation of C4‐2 cells* in vitro

We further evaluated the effects of exogenous PSA on C4‐2 cell proliferation using recombinant PSA protein (AbD Serotec, USA).^13^ As shown in Figure 2B, cells in groups under treatment with 500 ng/mL of PSA and 1000 ng/mL of PSA were accelerated in growth at 72 hours and 96 hours, compared with cells in control group. The data suggest that exogenous PSA is sufficient to promote PrCa cell proliferation.

### 3.4 Lo*ss of PSA expression attenuates migration and invasion of C4‐2 cells* in vitro

A previous study reported that transfection with PSA promoted migration rather than invasion of PrCa PC‐3 cells.^6^ To validate the effects of PSA in metastasis in vitro, we performed the Transwell cell invasion or migration assay with or without Matrigel Matrix to detect the changes in migration or invasion of PrCa cells under different PSA levels. Compared with the control group (shNC) (610.61 ± 46.46), the migration of Monoclone6 (391.23 ± 18.14) and Monoclone9 (360.74 ± 44.47) cells decreased by 35.93% (*P* < .001) and 40.92% (*P* < .001), respectively (Figure 2C); meanwhile, the invasion of Monoclone6 and Monoclone9 decreased by 62.27% (*P* = .001) and 66.98% (*P* = .001), respectively (Figure 2D). The results indicate that PSA is required for the migration and invasion of C4‐2 cells.

### 3.5 PSA knockdown promotes the apoptosis of C4‐2 cells in vitro

Flow cytometry assay was conducted to measure the apoptosis of C4‐2‐derived cells. The Quad 2/(Quad1 + Quad2 + Quad3 + Quad4) value, that is the proportion of late apoptotic/necrotic cells in all cells, was used to reflect apoptosis rate of cells for comparison (Figure 2E). Compared with the apoptosis rate of shNC cells ((1.51 ± 0.45)%), the apoptosis rate of Monoclone6 ((4.48 ± 1.09)%) (*P* = .039) and Monoclone9 ((6.55 ± 2.09)%) (*P* = .004) was significantly higher, suggesting that PSA knockdown promotes cell apoptosis.

### 3.6 *Loss of PSA expression slowed down tumorigenesis and inhibited the metastasis of C4‐2 cells* in vivo

**Figure 3:**
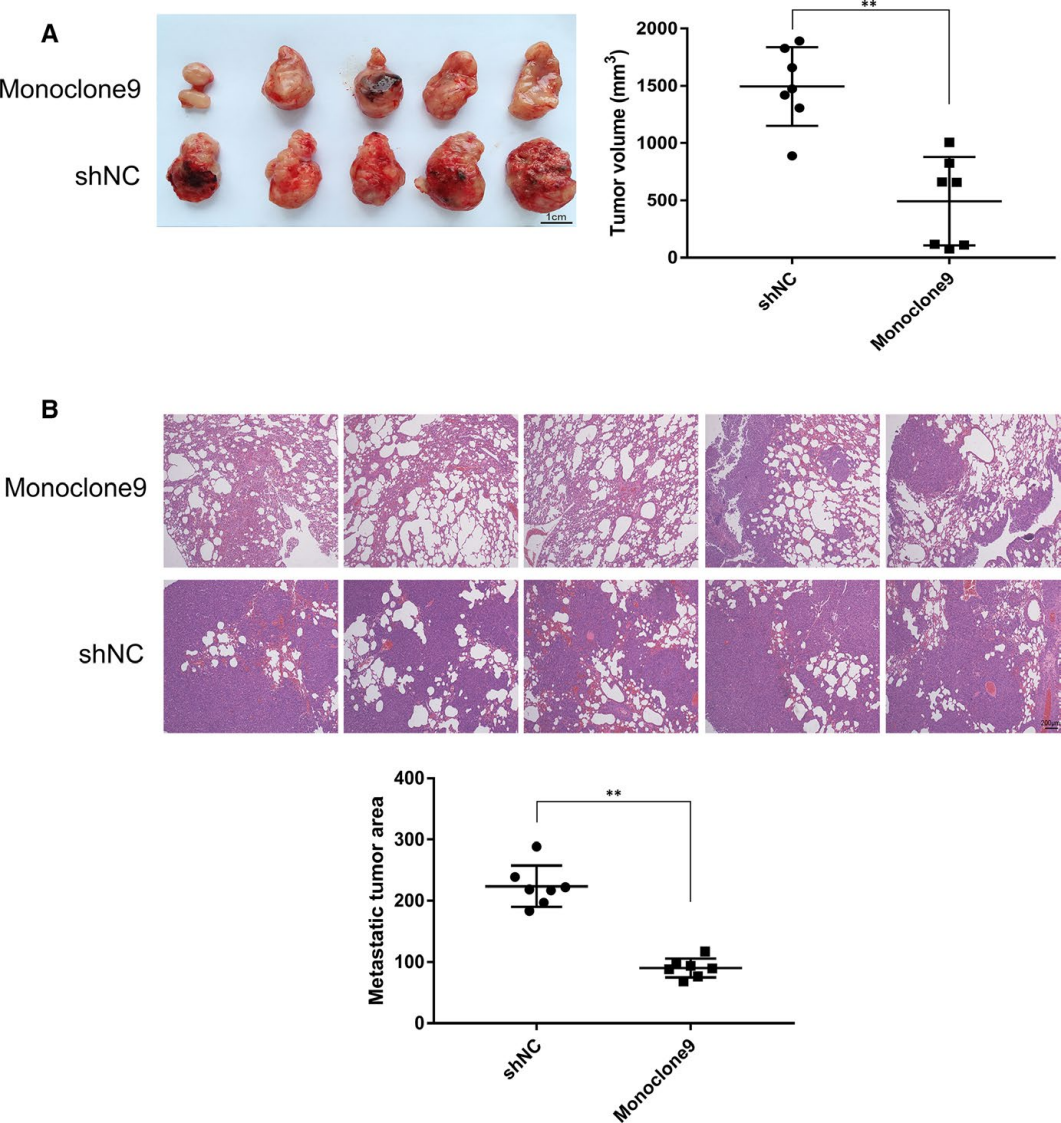
Effects of decreased PSA on tumorigenesis and metastasis of C4‐2 cells in vivo. A, Inhibited PSA expression obviously slowed down tumorigenesis (*P* < .001). n = 7. Scale bar = 1 cm. B, Interfered PSA shrank metastatic tumour area in mice lungs (*P* < .001), with treatment of HE staining. n = 7. Scale bar = 200 μm. ** shows *P* < .01

To evaluate the impact of reduced PSA on PrCa in vivo, we established murine models mirroring tumorigenesis and metastasis of C4‐2 cells in vivo. As shown in Figure 3, Figures S1 and S2, Monoclone9 groups showed minor tumour volumes (493.16 ± 385.51 mm^3^; *P* < .001) and smaller metastatic tumour area (*P* < .001) than shNC groups respectively, suggesting that loss of PSA expression delayed growth of C4‐2 cells in vivo and distant spreading to mice lung.

### 3.7 iTRAQ‐based LC‐MS/MS and hierarchical cluster analysis

To understand the proteomic change induced by PSA alteration, three samples of C4‐2‐derived shNC (control group) or Monoclone9 (test group) cells were subjected to iTRAQ‐based LC‐MS/MS analysis. Totally, 6822 proteins (Table S1) were detected, and 1061 of 6822 proteins were filtered in by the *P* value less than .05 (*P* < .05). Among these 1061 proteins, there were 121 proteins filtered in by fold change above 1.2 (fold change > 1.2). Among these 121 proteins, 67 proteins were up‐regulated while 54 proteins were down‐regulated.

**Figure 4:**
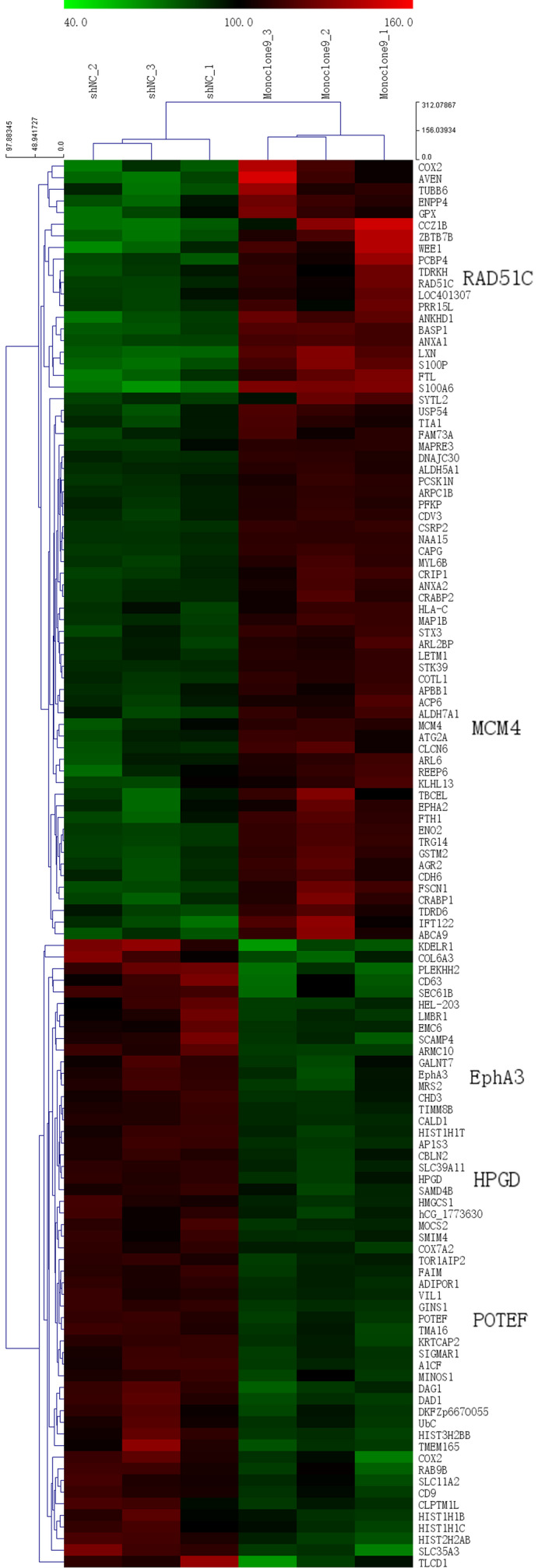
Heatmap based on Hierarchical cluster analysis of 121 differentially expressed proteins. Red denotes a high abundance of a protein, while green denotes a low abundance of the protein. Samples and proteins were clustered stepwise based on the homogeneity and similarity

A heatmap (Figure 4) of hierarchical cluster analysis was generated according to the abundance of 121 differentially expressed proteins (fold change > 1.2, *P* < .05), which showed visually obvious expression changes of these proteins due to the knockdown of PSA. Three replicates in either control group or test group were clustered first, indicating biological replicate samples were homologous.

### 3.8 GO and KEGG enrichment analysis

The 121 proteins differentially expressed were subjected to annotation and functional enrichment using ClueGO^9^ and CluePedia ^10^ plugins within Cytoscape based on GO and KEGG databases.

**Figure 5:**
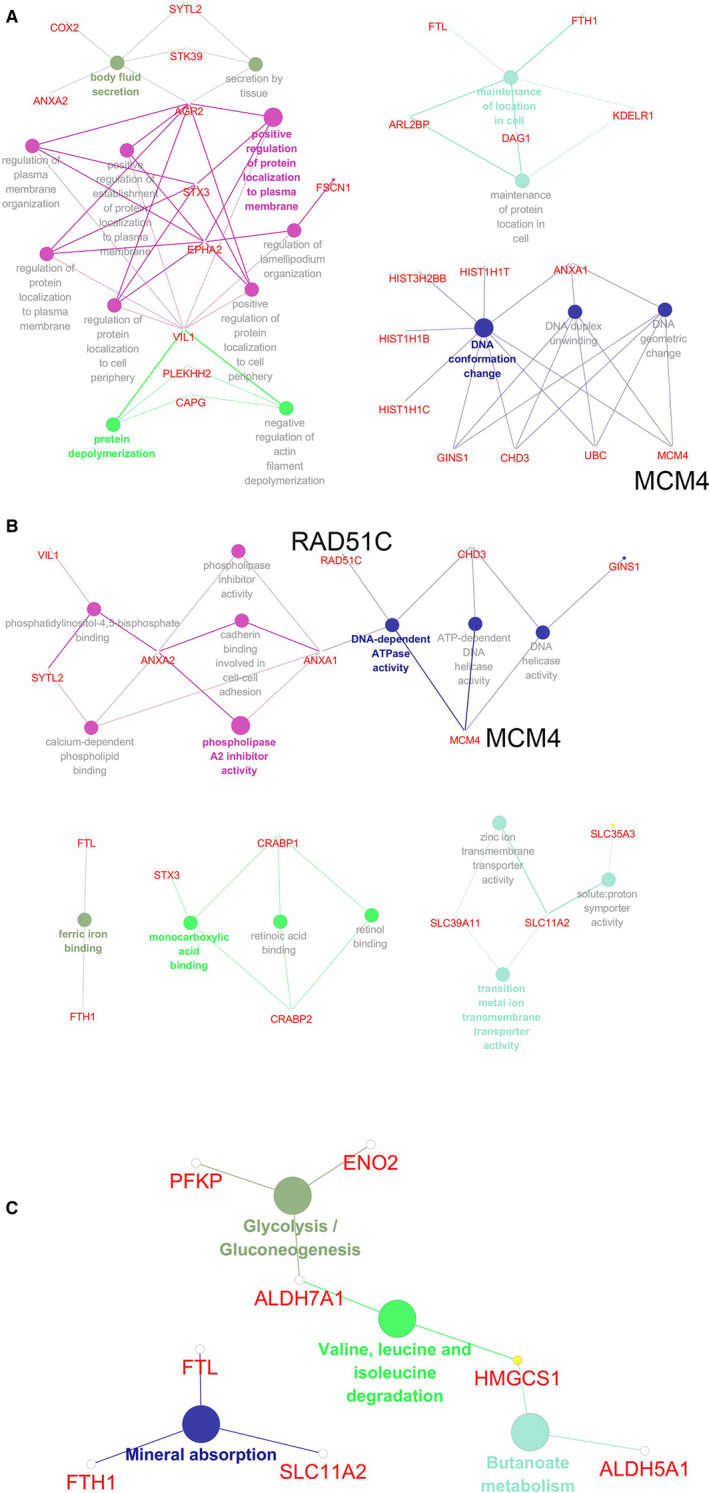
Enrichment and functional annotation by Gene Ontology (GO) and Kyoto Encyclopedia of Genes and Genomes (KEGG). A, GO Biological Process (BP) Classification presenting terms and relevant proteins. Nodes with bigger sizes denote GO BP terms; ones with smaller sizes denote associated proteins. B, GO Molecular Function (MF) Classification presenting terms and relevant proteins. Nodes with bigger sizes denotes GO MF terms; ones with smaller sizes denotes associated proteins. C, KEGG pathways enriched and associated proteins. Nodes with bigger sizes denote KEGG Pathway terms; ones with smaller sizes denote associated proteins

As shown in Figure 5A and Table 1, the proteins were predominantly enriched into GO BP terms including DNA conformation change, depolymerization, secretion, maintenance or regulation of location. The enriched GO MF terms included DNA‐related enzyme activity, phospholipase inhibitor activity, molecule binding and transporter activity (Figure 5B and Table 2).

**Table 1:** Biological process (BP) terms of Gene Ontology (GO) enriched and associated differentially expressed proteins

**GO ID**	**GO term**	***P* value**	**Protein identified**
GO:0 032 941	Secretion by tissue	.0022	[AGR2↑, STK39↑, SYTL2↑]
GO:0 007 589	Body fluid secretion	.0007	[AGR2↑, ANXA2↑, COX2↓, STK39↑, SYTL2↑]
GO:0 051 261	Protein depolymerization	.0244	[CAPG↑, PLEKHH2↓, VIL1↓]
GO:0 030 835	Negative regulation of actin filament depolymerization	.0024	[CAPG↑, PLEKHH2↓, VIL1↓]
GO:0 051 651	Maintenance of location in cell	.0008	[ARL2BP↑, DAG1↓, FTH1↑, FTL↑, KDELR1↑]
GO:0 032 507	Maintenance of protein location in cell	.0238	[ARL2BP↑, DAG1↓, KDELR1↓]
GO:0 071 103	DNA conformation change	.0001	[ANXA1↑, CHD3↓, GINS1↓, HIST1H1B↓, HIST1H1C↓, HIST1H1T↓, HIST3H2BB↓, MCM4↑, UBC↓]
GO:0 032 392	DNA geometric change	.0003	[ANXA1↑, CHD3↓, GINS1↓, MCM4↑, UBC↓]
GO:0 032 508	DNA duplex unwinding	.0002	[ANXA1↑, CHD3↓, GINS1↓, MCM4↑, UBC↓]
GO:1 903 729	Regulation of plasma membrane organization	.0018	[AGR2↑, EPHA2↑, STX3↑, VIL1↓]
GO:1 904 377	Positive regulation of protein localization to cell periphery	.0002	[AGR2↑, EPHA2↑, STX3↑, VIL1↓]
GO:1 902 743	REGULATION of lamellipodium organization	.0026	[EPHA2↑, FSCN1↑, VIL1↓]
GO:1 904 375	Regulation of protein localization to cell periphery	.0013	[AGR2↑, EPHA2↑, STX3↑, VIL1↓]
GO:0 090 004	Positive regulation of establishment of protein localization to plasma membrane	.0012	[AGR2↑, EPHA2↑, VIL1↓]
GO:1 903 076	Regulation of protein localization to plasma membrane	.0011	[AGR2↑, EPHA2↑, STX3↑, VIL1↓]
GO:1 903 078	Positive regulation of protein localization to plasma membrane	.0001	[AGR2↑, EPHA2↑, STX3↑, VIL1↓]

Note

↑ up‐regulated; and ↓ down‐regulated.

**Table 2:** Molecular function (MF) terms of Gene Ontology (GO) enriched and associated differentially expressed proteins

**GO ID**	**GO term**	***P* value**	**Protein identified**
GO:0 008 199	Ferric iron binding	.0022	[FTH1↑, FTL↑]
GO:0 019 841	Retinol binding	.0050	[CRABP1↑, CRABP2↑]
GO:0 033 293	Monocarboxylic acid binding	.0080	[CRABP1↑, CRABP2↑, STX3↑]
GO:0 001 972	Retinoic acid binding	.0050	[CRABP1↑, CRABP2↑]
GO:0 015 295	Solute: proton symporter activity	.0119	[SLC11A2↓, SLC35A3↓]
GO:0 046 915	Transition metal ion transmembrane transporter activity	.0256	[SLC11A2↓, SLC39A11↓]
GO:0 005 385	Zinc ion transmembrane transporter activity	.0081	[SLC11A2↓, SLC39A11↓]
GO:0 003 678	DNA helicase activity	.0057	[CHD3↓, GINS1↓, MCM4↑]
GO:0 008 094	DNA‐dependent ATPase activity	.0018	[ANXA1, CHD3↓, MCM4↑, RAD51C↑]
GO:0 004 003	ATP‐dependent DNA helicase activity	.0245	[CHD3↓, MCM4↑]
GO:0 005 544	Calcium‐dependent phospholipid binding	.0031	[ANXA1↑, ANXA2↑, SYTL2↑]
GO:0 004 859	Phospholipase inhibitor activity	.0026	[ANXA1↑, ANXA2↑]
GO:0 098 641	Cadherin binding involved in cell‐cell adhesion	.0050	[ANXA1↑, ANXA2↑]
GO:0 019 834	Phospholipase A2 inhibitor activity	.0002	[ANXA1↑, ANXA2↑]
GO:0 005 546	Phosphatidylinositol‐4,5‐bisphosphate binding	.0059	[ANXA2↑, SYTL2↑, VIL1↓]

Note

↑ up‐regulated; and ↓ down‐regulated.

Figure 5C and Table 3 indicated the enriched KEGG pathways with relevant proteins. There were four KEGG pathways being substantially enriched: glycolysis/gluconeogenesis, valine, leucine and isoleucine degradation, butanoate metabolism and mineral absorption, suggesting a potential regulation of genome stability and metabolism by PSA.

**Table 3:** Kyoto Encyclopedia of Genes and Genomes (KEGG) pathways enriched and associated differentially expressed proteins

**GO ID**	**GO term**	***P* value**	**Protein identified**
GO:0000010	Glycolysis/Gluconeogenesis	.0073	[ALDH7A1↑, ENO2↑, PFKP↑]
GO:0 000 280	Valine, leucine and isoleucine degradation	.0331	[ALDH7A1↑, HMGCS1↓]
GO:0 000 650	Butanoate metabolism	.0120	[ALDH5A1↑, HMGCS1↓]
GO:0 004 978	Mineral absorption	.0036	[FTH1↑, FTL↑, SLC11A2↓]

Note

↑ up‐regulated; and ↓ down‐regulated.

### 3.9 Protein‐protein interaction (PPI) network by STRING and cytoHubba analyses

**Figure 6:**
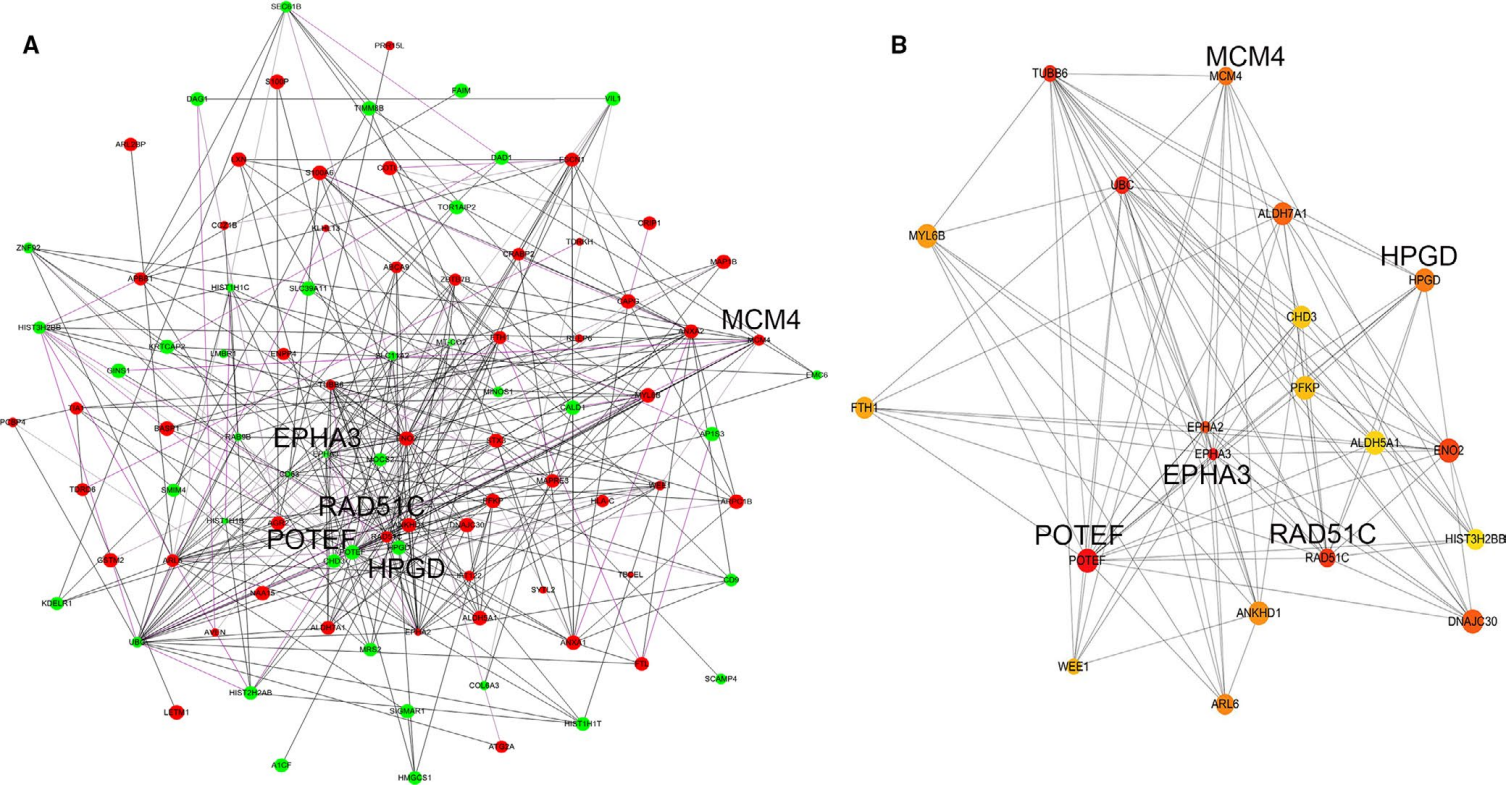
Protein‐protein interaction (PPI). A, PPI network of 121 differentially expressed proteins generated using Search Tool for the Retrieval of Interacting Genes (STRING) database and Cytoscape. Each node represents a protein, and each edge represents an interaction. Red and green nodes indicate up‐regulated proteins and down‐regulated proteins. The node size depends on the P value representing differentiation, that means larger the size is, smaller the P value is. B, PPI subnetwork of top 20 Hubba nodes ranked by maximum neighbourhood component (MCC) method using the cytoHubba plugin in Cytoscape. Each node represents a protein, and each edge represents an interaction. The node colour varies from red to yellow with a MCC rank

To explore the association of 121 differentially expressed proteins and further discover potential candidate proteins that may function in the biological process of PrCa, STRING database and cytoHubba were sequentially utilized in data mining. The PPI network was firstly generated as shown in Figure 6A, one node denoting one protein and one edge denoting one potential correlation between every two proteins. After importing the STRING PPI network into Cytoscape, top 20 nodes were further selected and ranked by MCC in cytoHubba, which were mapped into a subnetwork (Figure 6B). These 20 proteins were considered as the most important part of the STRING network according to MCC topological analysis. Among them, 6 proteins were down‐regulated, and the other 14 proteins were up‐regulated. Five proteins were identified.

### 3.10 Validation of MCM4 expression in PrCa and para‐cancerous tissues

**Figure 7:**
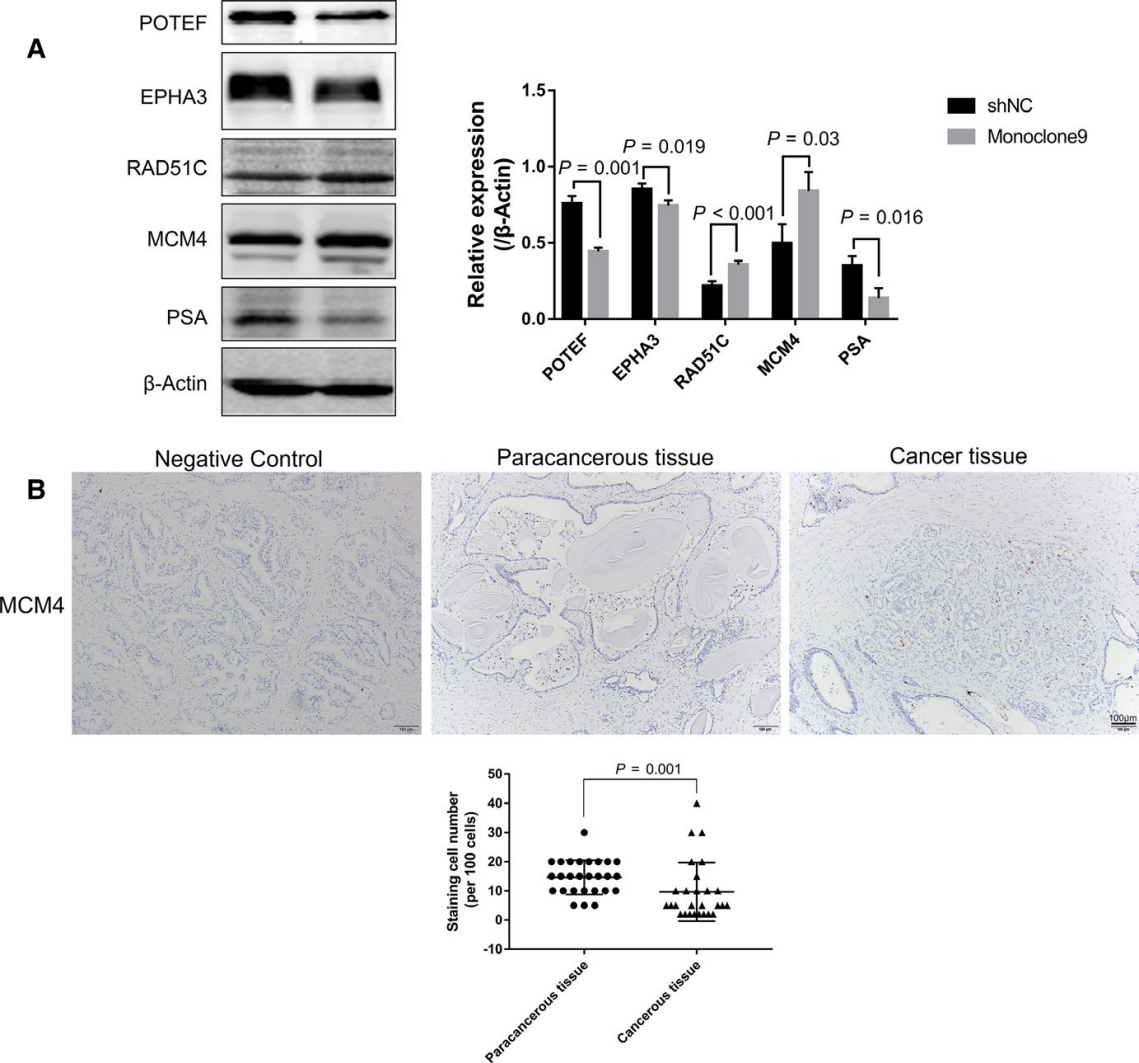
Up‐regulated MCM4 expression induced by reduction of PSA and expression of MCM4 in prostate cancer tissue and para‐cancerous tissue. A, Once silencing of PSA, MCM4 and RAD51C were up‐regulated in C4‐2 cells, while POTEF and EPHA3 were down‐regulated. B, Expression level of MCM4 in para‐cancerous tissue is higher than that in para‐cancerous tissue. Scale bar = 100 μm

Five proteins identified were listed as follows: POTE ankyrin domain family member F (POTEF), EPH receptor A3 (EPHA3), DNA repair protein RAD51 homolog 3 (RAD51C), minichromosome maintenance complex component 4 (MCM4) and hydroxyprostaglandin dehydrogenase 15‐(NAD) (HPGD). Among them, MCM4, RAD51C, EPHA3 and POTEF were subjected to the verification of the efficiency of proteome analysis. Western blot assay was used to validate changes of MCM4, RAD51C, EPHA3 and POTEF induced by loss of PSA in C4‐2 cells. As shown in Figure 7A, MCM4 expression (*P* = .03) and RAD51C expression (*P* < .001) rose up, while POTEF expression (*P* = .001) and EPHA3 expression fell down (*P* = .019), because of the decreased PSA expression. And to investigate the underlying role of MCM4 in PrCa, the level of MCM4 in PrCa cancerous tissue and para‐cancerous tissue was measured using IHC. As shown in Figure 7B, MCM4 located predominantly in prostate gland epithelial cells. The staining cell number per 100 epithelial cells was defined as the parameter of evaluation of staining result, and the evaluation work was performed by two pathologists. The MCM4 expression is lower in para‐cancerous tissue than in cancerous tissue (*P* = .001). These data confirmed the reliability of our proteomic results.

## 4 DISCUSSION

The failure of current therapeutic approaches to prevent PrCa from recurrence or evolving into a castration‐resistant state with a high mortality rate promotes various research programmes exploring the pathological mechanism of the initiation and development of PrCa. As a serine proteinase and biomarker, PSA plays a pivotal role in the pathological process of PrCa, although previous studies achieved heterogeneous conclusions as to either positive or negative role in PrCa.^3^, ^5^, ^6^, ^7^ However, rare researches could explain the mechanism through which PSA functions in the development and progression of PrCa.

In the present study, a gene specific shRNA construct was used to knock down PSA expression in CRPC C4‐2 cells. To acquire C4‐2 cells homogeneously expressing decreased PSA, we performed the screening of monoclonal colonies for subsequent experiments. This study confirms that PSA facilitates growth and metastasis of PrCain vitro and in vivo, and attenuates apoptosis of PrCa cells in vitro. These results consolidate the hypothesis that PSA is a key molecule in pathology of PrCa and emphasize the need for investigating the underlying mechanism whereby PSA plays the role.

Proteomic analysis could gain an insight into a proteome‐wide change of proteins induced by PSA. Bindukumar et al performed a proteomic analysis for prostate cells treated by free PSA^14^; alterations to the LNCaP proteome in conditioned media induced by down‐regulated PSA were measured using a mass spectroscopy‐based approach.^3^ We first measured the proteome‐wide alteration of in CRPC C4‐2 cell lysates using iTRAQ‐based LS‐MS/MS and performed a systematical bioinformatics analysis of differentially expressed proteins.

The hierarchical cluster analysis indicated that the quantified data were replicable and reduced PSA induced a wide change of proteins. Combination with the PPI subnetwork with GO/KEGG analysis further contributes to a better insight into changes induced by PSA. Using these methods, we chose five candidate proteins which may be under regulation by PSA, including POTEF, EPHA3, RAD51C, MCM4 and HPGD. POTEF, a membrane glycoprotein, was speculated that the enhanced galactosylation of it may contribute to the early step of TNBC metastasis.^15^ In combination with the alteration of metastasis in PSA‐silenced cells, it is speculated that PSA may regulate the expression or modification of TNBC. As a receptor tyrosine kinase and frequently overexpressed on the tumour‐initiating cell population in glioma, EPHA3 modulates mitogen‐activated protein kinase signalling to maintain glioblastoma cells in a less differentiated state, and once it is knocked down, tumorigenic potential of tumour cells is weaken.^16^ Charmsaz et al^17^ discovered that anti‐EPHA3 monoclonal antibody (mAb) could attenuate the growth of EPHA3‐expressing leukaemic xenografts. These results are consistent with the finding in the present study. RAD51C is essential for DNA repair by homologous recombination.^18^ RAD51C deficiency accumulates somatic mutation, which leads to genomic instability, and finally promotes cancer.^19^ In addition, loss of RAD51C accelerates tumour progression lacking of Trp53.^20^ These studies suggest that up‐regulated RAD51C by knockdown of PSA may attenuate CRPC progression. MCM4 is a component of the minichromosome maintenance complex (MCM2‐7 helicase complex) essential for normal DNA replication and genome stability^21^ which is involved in initiating cancer,^22^ and is required for terminal NK cell maturation,^23^ while NK cells shows antitumoural activity in the mouse model.^24^ The present study indicated that MCM4 is enriched in GO BP terms DNA geometric change and DNA duplex unwinding, as well as GO MF terms DNA helicase activity and DNA‐dependent ATPase activity. Increased MCM4 may indicate the negative regulation by PSA expression and further hint at an elevated immunoreactivity aiming to tumour and reinforced genome stability. HPGD, hydroxyprostaglandin dehydrogenase 15‐(NAD), was found to be abundantly expressed in highly metastatic breast cancer cells, while knockdown of HPGD attenuated aryl hydrocarbon receptor signalling and induced mesenchymal‐epithelial transition.^25^ In our study, PSA silencing induced down‐regulation of HPGD and attenuated migration and invasion, which indicated that PSA may exert an influence on PrCa cells via regulating epithelial‐mesenchymal transition (EMT).

In addition of the down‐regulation of these five proteins, the present study also found some down‐regulated proteins. HIST3H2BB is a member of the histone H2B family. S14 of histone H2B can be bound and phosphorylated by HIPK2, while loss of H2B‐S14^P^ induced by HIPK2 depletion can prevent cell cleavage and result in tetra‐ and polyploidization. Cytokinesis defects and cell proliferation could be rescued by restoration of wild‐type HIPK2 activity or expression of a phosphomimetic H2B‐S14D derivative, suggesting H2B‐S14^P^ is essential for a faithful cytokinesis.^26^ The present study found that PSA silencing decreased HIST3H2BB expression and proliferation of C4‐2 cells suggesting that PSA may exert an influence on proliferation of PrCa via modulating cytokinesis.

Phosphofructokinase, platelet (PFKP), a major isoform of cancer‐specific phosphofructokinase‐1 (PFK‐1) enriched in glycolysis/gluconeogenesis pathway, plays a duel role in cancer cell survival.^27^ Most cancer cells acquire energy mainly via a high rate of aerobic glycolysis in nourished condition, in which PFKP is the first rate‐limiting enzyme.^27^, ^28^ But in resource‐limited condition, such as glucose deficiency or detachment of tumour cells from their natural extracellular matrix (ECM), in particular in the process of metastasis, tumour cells are under metabolic stress.^27^, ^29^ Metabolic stress can suspend aerobic glycolysis via suppressing PFKP activity and direct glucose flux into the pentose phosphate pathway (PPP); on the other hand, NADPH of PPP could resist metabolic stress and enable survival of tumour cells, while knockdown of PFKP rescued cell death caused by loss of Snail under metabolic stress.^27^ In the present study, knockdown of PSA induced the rising up of PFKP, suggesting that PSA may maintain the survival of PrCa cells via modulating glucose flux.

Hydroxymethylglutaryl‐CoA synthase‐1 (HMGCS1), an enzyme involved in lipid metabolic process,^30^ is classified into two KEGG pathways: valine, leucine and isoleucine degradation, and butanoate metabolism in KEGG pathway. HMGCS1 was reported to be over twofold higher in a non‐small‐cell lung cancer Abraxane‐resistant cells than Abraxane‐sensitive, which indicated involvement of HMGCS1 in drug resistance.^30^ In the present study, HMGCS1 was decreased after reduction of PSA, which implies that PSA may participate in the resistance to androgen deprivation therapy.

The up‐regulated MCM4 expression and RAD51C expression, as well as the down‐regulated POTEF expression and EPHA3 expression, were verified caused by change of PSA expression, which suggests that PSA may mediate the expression of MCM4, RAD51C, POTEF and EPHA3. The differentially expressed MCM4 in cancerous and para‐cancerous tissue implies that MCM4 may exert an influence on tumorigenesis of PrCa via interfering the genome stability. In all, PSA may regulate the tumorigenesis of PrCa via mediating the MCM expression in epithelial cells which is critical to DNA replication and genome stability.

In summary, iTRAQ‐based LC‐MS/MS was employed to analyse the proteomic differentiation caused by reduced PSA. The results suggested that PSA would exert influences on the development and progression of PrCa via modulating EMT, cell cleavage, glucose metabolism or lipid metabolism, especially genomic instability in which MCM4 may be involved. However, in view of the only one PrCa cell line applied in this study, the hypothesis remains to be further verified in experiments involving additional cell lines. In addition, further research should be undertaken to investigate the mechanism whereby PSA regulate POTEF, EPHA3, RAD51C, HPGD and especially MCM4. Even so, our study verifies PSA as a promoting factor in PrCa and unravels the potential mechanism triggered by PSA in the development and progression of PrCa.

## 5 CONCLUSIONS

We have confirmed that PSA exerts considerable influence on tumorigenesis and metastasis of PrCa via mediating proliferation, migration, invasion and apoptosis of PrCa cells. Besides, an iTRAQ‐based LC‐MS/MS was performed to detect the comprehensive molecular mechanism of PSA elucidating its role in tumorigenesis and metastasis of PrCa. In detail, PSA would modulate EMT, cell cleavage, glucose metabolism or lipid metabolism, especially genomic instability via mediating POTEF, EPHA3, HPGD, RAD51C, and especially MCM4. Further research should be performed to explore the mechanism of PSA involved in tumorigenesis and metastasis of PrCa, supporting the therapy target of PSA.

## CONFLICTS OF INTEREST

There were no potential conflicts of interest to be disclosed.

## AUTHOR CONTRIBUTION

**Haoyong Li:** Writing‐original draft (equal). **Zhe Ma:** Data curation (lead). **Zhifei Che:** Data curation (equal). **Qi Li:** Methodology (equal). **Jinfeng Fan:** Validation (equal). **Zhiyan Zhou:** Software (equal). **Yaoxi Wu:** Visualization (equal). **Yingxia Jin:** Visualization (equal). **Peiyu Liang:** Writing‐original draft (lead). **Xianping Che:** Writing‐original draft (equal).

## Supporting information

Figure S1

Figure S2

Table S1

## Data Availability

The data used to support the findings of this study are available from the corresponding author upon request.
