# Supplementary figures and images for: Comprehensive role of prostate‐specific antigen identified with proteomic analysis in prostate cancer
Source: J Cell Mol Med. 2020 Jul 27;24(17):10202–15. doi: 10.1111/jcmm.15634 (PMC7520270; doi:10.1111/jcmm.15634)

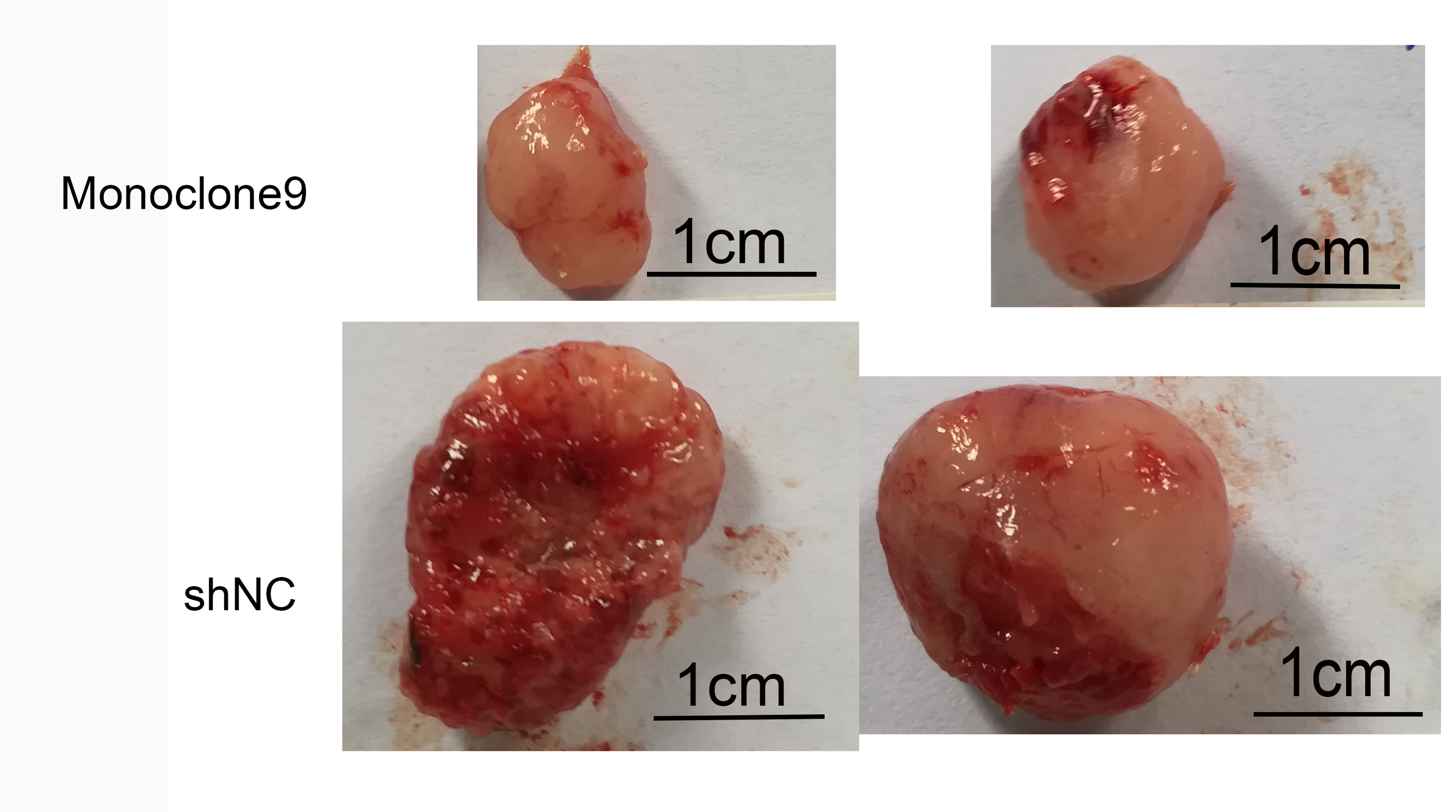

Supplement: Supplementary file 1 — Figure S1 [file JCMM-24-10202-s001.tif]

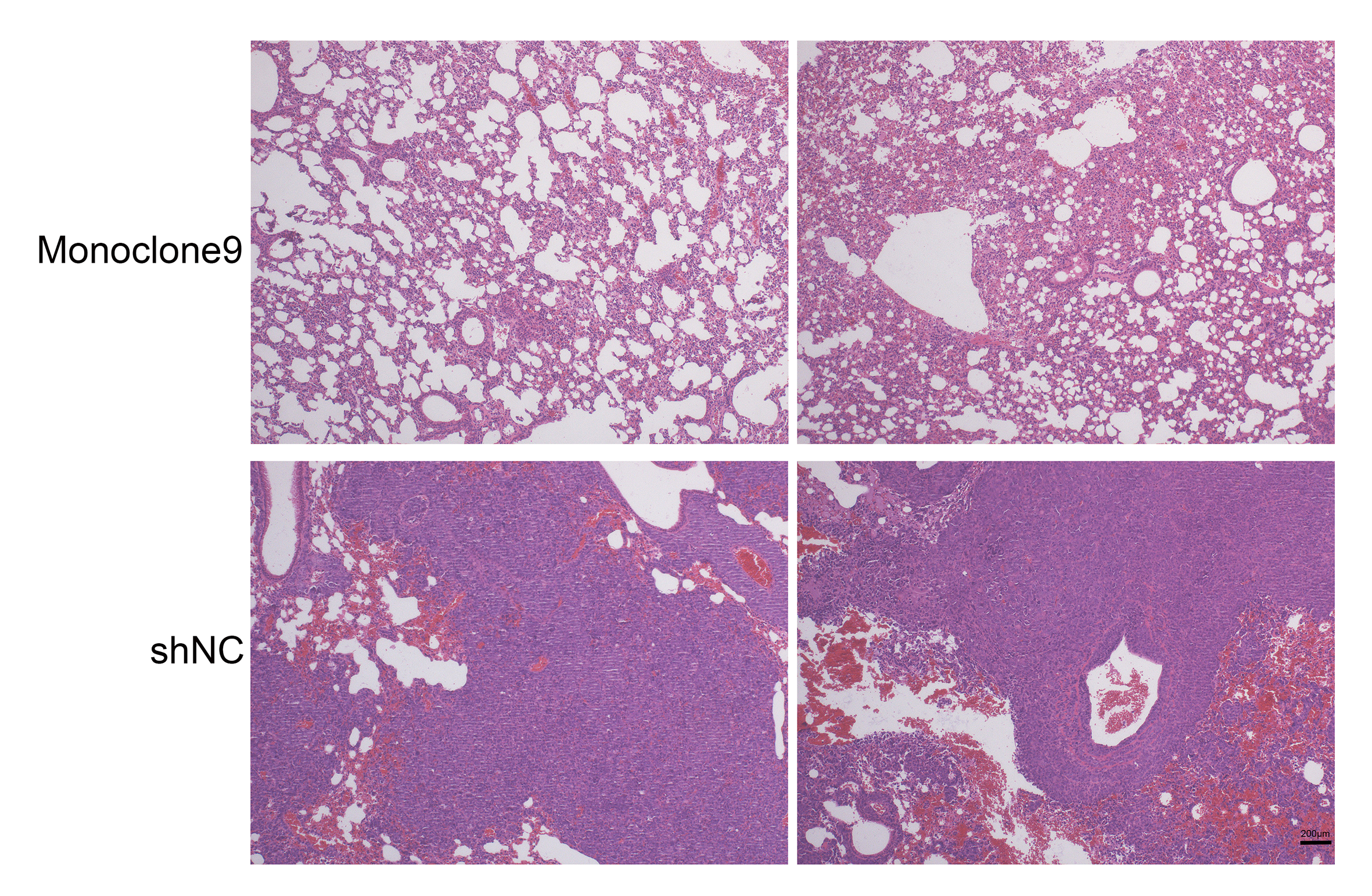

Supplement: Supplementary file 2 — Figure S2 [file JCMM-24-10202-s002.tif]
